# Is nature truly healing itself? Spontaneous remissions in Paroxysmal Nocturnal Hemoglobinuria

**DOI:** 10.1038/s41408-021-00582-5

**Published:** 2021-11-27

**Authors:** Carmelo Gurnari, Simona Pagliuca, Tariq Kewan, Waled Bahaj, Minako Mori, Bhumika J. Patel, Valeria Visconte, Jaroslaw P. Maciejewski

**Affiliations:** 1 Department of Translational Hematology and Oncology Research, Taussig Cancer Institute, Cleveland Clinic, Cleveland, OH USA; 2 Department of Biomedicine and Prevention, PhD in Immunology, Molecular Medicine and Applied Biotechnology, University of Rome Tor Vergata, Rome, Italy; 3 University of Paris, Paris, France

**Keywords:** Haematopoietic stem cells, Haematological diseases

**Dear Editor,**

Paroxysmal nocturnal hemoglobinuria (PNH) is considered to be curable only through the means of allogeneic stem cell transplantation (SCT) [1]. One of the many fascinating and scientifically instructive aspects of the pathogenesis of this disease is the unique possibility of its spontaneous remission with disappearance of PNH cell populations and abatement of clinical symptoms, a rare event which has always captivated the research community [2–4]. Due to the orphan nature of the condition, no clinical predictors have been identified so far as harbingers of this phenomenon estimated to occur in up to 15% (3–15% according to the different series) of PNH patients between 10 and 20 years from disease onset [3, 5]. In a classical scenario, exhaustion of the PNH clone (theory of “finite life span”) may be associated with reappearance of aplastic anemia (AA), in which the emergence of a PNH clone reflects a semi-maladaptive attempt of recovery [3]. Consequently, one could stipulate that a retraction of the PNH clone would have to be associated with a compensatory re-establishment of normal hematopoiesis should normal blood counts be maintained [6]. The latter hypothesis is supported by anecdotal cases of PNH following autologous SCT (ASCT), in which PNH expansion would be favored by either post-ASCT stem cell pool contraction or immune system derangements, with subsequent extinction of the PNH cell populations upon cessation of these supporting conditions [6, 7]. Environmental changes underlying PNH clone expansion/contraction may also provide support for the theory of the “neutral drift” which, by applying in silico models of Markovian stochastic dynamics, posits the absence of a selective advantage of *PIGA* mutant clones [8, 9]. Furthermore, the recent insights into the AA/PNH pathobiology shed light onto molecular underpinnings of polyclonal vs. oligoclonal hematopoiesis and their dynamics as to the possible acquisition over time of clonal hematopoiesis of indeterminate potential (CHIP) [10, 11]. Nevertheless, literature data on genetics of PNH spontaneous remission are scarce with to date only one case molecularly characterized by means of modern technologies (e.g., whole-exome sequencing) [2]. Herein, through the application of multiple next-generation sequencing (NGS) panels interrogating genes involved in PNH clonal evolution, we attempted to better discern the genomic mechanisms of its spontaneous remission.

In total, our study cohort included 92 patients with a PNH granulocyte clone size >20% and lactate dehydrogenase > x2.5 upper limit of normal with or without evidence of bone marrow failure (see also Supplemental Material) [12, 13]. Flow cytometry studies for determination of PNH granulocyte/red blood cell (RBCs) size and type (incomplete loss of CD59 i.e., type II vs. complete loss i.e., type III) were performed as previously described [12, 13]. Deep sequencing of *PIGA* gene was performed on whole blood DNA using multi-amplicon NGS with primers covering all coding exons [14]. NGS of 62 myeloid genes (Table S1) and HLA class I/II *loci* along with a newly developed in-house bioinformatic pipeline (Fig. S1) were used for the detection of somatic clonal events (see also Supplemental Material) [15, 16].

Among 92 patients with a diagnosis of PNH (M:F ratio 0.88, median age 38, range 9–84) 41% were classified as primary PNH (pPNH), 49% were secondary to AA (sPNH) and 10% were classified as AA/PNH overlap syndrome. Overall, patients were clinically followed-up for a median time of 68 months (range, 2–339). Median granulocyte clone size was 73% (range, 22–99) with the majority of cases (80%) being classified as having a type III dominant RBCs clone while 20% having a type II. Within this cohort, a total of 3 patients experienced spontaneous remission at a median time of 20 years (range, 1–22) from PNH diagnosis (Fig. 1).

**Fig. 1:**
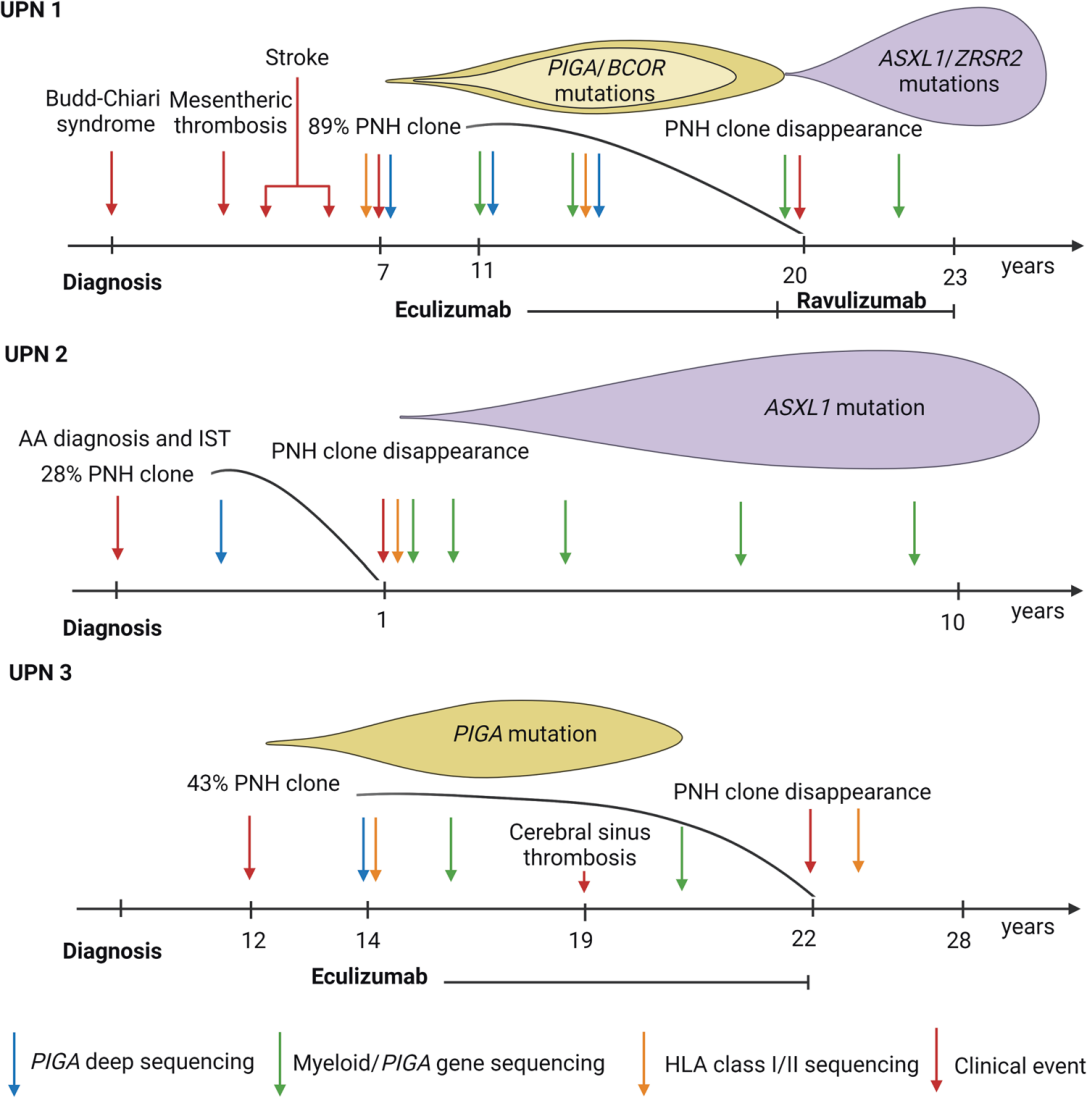
Timeline of events for patients with PNH undergoing spontaneous remission. The figure showcases the disease course of the presented patients (UPN1-3) discussed in the main text as to clinical events (e.g., thrombotic complications, treatments), flow cytometric and molecular (*PIGA*, myeloid, HLA panels) data.

UPN1 was a 69-year-old male diagnosed with pPNH at the age of 46 after an episode of deep vein thrombosis. He had been managed with prednisone, transfusions and anticoagulation because of recurrent thrombotic episodes. Once available, he was started on eculizumab and later continued on ravulizumab. His initial flow cytometry study revealed the presence of a type III RBCs clone of 40% and a granulocyte clone of 89%. After 9 years of anticomplement therapy, the patient’s clone started a slow decrease and the most recent flow cytometric study revealed a granulocyte clone of 0.02%. Molecular analysis performed at the time of eculizumab start showed a PNH-dominant mutational configuration by adjusted variant allelic frequency (VAF) with *PIGA* deletion (p.94_95del; adjusted VAF 29%) and a *BCOR* nonsense (p.Y1446X; VAF 13%). No HLA class I/II mutations were found in two longitudinal samples collected 1 year before and after eculizumab start. However, at the last sequencing performed after the complete disappearance of the PNH cell population, the patient developed *ASXL1* (p.E635Rfs*; VAF 26%) and *ZRSR2* (p.E120Gfs*; adjusted VAF 21%) mutations with a co-dominant configuration along with retraction of the previous *PIGA* lesion. No decrease in blood counts was noted.

UPN2 was a 58-year-old male initially diagnosed with severe AA at the age of 48 and treated with ATG + cyclosporine. At that time, he had a co-existing PNH granulocyte clone of 28% (AA/PNH overlap syndrome). During the year following immunosuppressive treatment, his PNH clone rapidly dropped to 1% and since then has been consistently below 1% (last 0.1%). Therefore, the patient has never received anticomplement therapy. At the time of PNH clone retraction, no HLA class I/II or myeloid driver mutations were found and *PIGA* mutations were not detectable. However, longitudinal molecular studies performed after disappearance of PNH cell population revealed the acquisition of *ASXL1* p.Q512X mutation at an initial VAF of 23%, which doubled (45%) at last follow-up 5 years later while normal counts persisted.

UPN3 was a 59-year-old lady diagnosed with pPNH at the age of 30. She had a granulocyte clone as high as 43% with a type II RBCs clone of 17% and a typical *PIGA* splice site c.981 + 1 G > A mutation (adjusted VAF 15%). She was initially treated with transfusions and steroids and her course was complicated by a cerebral venous sinus thrombosis. Patient was eventually given eculizumab and her PNH clone started decreasing until it vanished (last 0.04%) after 8 years from treatment start. Analysis of samples prior to and after PNH disappearance did not show any HLA class I/II nor myeloid driver gene mutations with absence of *PIGA* alterations at last sequencing. At last follow-up 6 years from eculizumab cessation, the patient persisted with normal blood counts.

Of note, in our patients with extinction of PNH cell populations anticomplement treatment cessation was never followed by recrudescence of the hemolytic process, nor there was evidence for recurrent AA.

PNH spontaneous remissions are rare events and still constitute an intriguing conundrum for the attending physicians. Through an extensive molecular study of longitudinal clonal dynamics, we demonstrated that in addition to be replaced by polyclonal (i.e., normal) hematopoiesis, *PIGA* clones may be swept by CHIP-like lesions in myeloid genes (e.g., *ASXL1*), possibly characterized by improved fitness advantage in a process of Darwinian selection. In some cases, the retraction or even the exhaustion of a PNH clone may be the consequence of the restoration of normal hematopoiesis, as often observed in patients with AA receiving successful immunosuppressive treatment (as exemplified by UPN2) [17]. Alternatively, myeloid clones may represent the epiphenomenon of intricate dynamics of clonal succession, which affect a substantial proportion of PNH patients despite not driving any clear clinical outcome [10].

In summary, although the meaning of CHIP-like lesions in AA/PNH remains to be fully elucidated, our results highlight the need for a watchful evaluation of clinically-apparent PNH remissions which, in some instances, can be replaced by conditions (e.g., CHIP) with a potential higher risk of malignant transformation. Future studies providing a magnified resolution of the molecular architecture of PNH clonal evolution at individual cell level (e.g., single-cell DNA sequencing) are warranted in order to potentially backtrack minor clones present prior to the disappearance of PNH clones, possibly presenting an increased fitness advantage in the “war of clones” of AA/PNH immunodynamics.

## Supplementary information

Supplemental Materials

Checklist

## Data Availability

Requests for additional information should be sent to the corresponding author.
