# Supplementary material for: Is nature truly healing itself? Spontaneous remissions in Paroxysmal Nocturnal Hemoglobinuria
Source: Blood Cancer J. 2021 Nov 27;11(11):187. doi: 10.1038/s41408-021-00582-5 (PMC8627504; doi:10.1038/s41408-021-00582-5)
Supplement: Supplementary file 1 — Supplemental Materials [file 41408_2021_582_MOESM1_ESM.docx]

**Supplemental Materials**

**Patients.** Specimens were obtained with written informed consent following the Declaration of Helsinki and in accordance to ethics committee approvals of The Cleveland Clinic Foundation. In total, our study cohort included 92 patients with a diagnosis of paroxysmal nocturnal hemoglobinuria (PNH) (PNH granulocyte clone size >20% and lactate dehydrogenase >x2.5 upper limit of normal) and aplastic anemia/PNH overlap syndrome when a co-existing diagnosis of bone marrow failure was evident as per previously published criteria. (1-3)

**Flow Cytometry.** A 5-color cocktail (CD15-V450, CD45-PC7, CD64-APC, CD157-PE, FLAER-Alexa 488) to identify granulocytes with variable or no expression of glycosylphosphatidylinositol (GPI)-linked proteins was used to detect and quantify PNH granulocytes clone size according to previous studies (Alternate protocol 2: “Simultaneous High‐Sensitivity Detection of PNH Neutrophils and Monocytes Using FLAER and CD157‐Based 5‐Color Assay”(4, 5)) while CD59-PE (Invitrogen, MHCD5904) and CD235a-FITC (Beckman Coulter (BC), IM2212U) staining was used for determination of PNH red blood cells as previously described.(1, 2) Cells were acquired on a FC500 or XL-MCL (Beckman Coulter) cytometer.

**Genetic studies.**

*Myeloid panel.* Patients with PNH were routinely tested for somatic mutations when clinically indicated and/or to rule out the possibility of evolution to a myeloid disorder using the diagnostic panel currently available at The Cleveland Clinic Foundation. When available, samples from our bio-repository were also used to study longitudinal clonal dynamics with the same gene panel described here. Briefly, DNA extracted from peripheral blood or bone marrow specimens was subjected to nested multiplex PCR-based target enrichment. Coding and non-coding regions of 62 genes (see **Supplemental Table 1**) were amplified and sequenced on an Illumina instrument (San Diego, CA) with paired end, and 150x2 cycle reads. A customized bio-informatic analytical pipeline was used to map reads to the reference human genome (Genomic Build GRCh37/hg19). During internal validation, this test delivered an average of >500X coverage and >98% of targeted regions showed over 100X coverage. The limit of detection of this test is 1% for the JAK2^V617F^ variant and 5% for other variants. Variants were classified according to the guidelines of the Association for Molecular Pathology guidelines.(6) Moreover, a bio-analytic pipeline developed in-house was used to call somatic/germline mutations using sequences derived from controls and mutational databases such as dbSNP138, 1000 Genomes or ESP 6500 database, and Exome Aggregation Consortium (ExAC). (7-11)

*Deep Targeted PIGA Sequencing.* Primers covering all exons of *PIGA* gene were designed to amplify genomic DNA for sequencing on an Illumina MiSeq sequencer according to our previous report.(12) Mutations were annotated using Annovar and their somatic status was called using an in-house bio-analytic pipeline, by comparison with sequenced healthy subjects and mutational databases such as dbSNP138, 1000 Genomes, ESP 6500, Exome Aggregation Consortium (ExAC) and gnomAD. (7-11)

*HLA sequencing and analysis.* HLA targeted sequencing was performed with TruSight HLA v2 (Illumina, San Diego CA). In brief (**Supplemental Figure 1**), 11 HLA loci (Class I HLA-A, B, and C Class II HLA-DRB1/3/4/5, HLA-DQA1, HLA-DQB1, HLA-DPA1, and HLA-DPB1) were amplified with a long-range PCR. After amplification, a transposon-based DNA tagmentation was applied to generate DNA amplicons, via DNA fragmentation and addition of adapter sequences. Further PCR steps provided sequence adapters and indexing primers to generate DNA libraries which were then loaded directly onto a MiSeq system for sequencing.

After obtaining a full 8-digit typing with NovoHLA (Novocraft Biotechnologies, Malaysia), a recently implemented allelic inference pipeline, paired-end reads from targeting sequencing were directly aligned on a per-patient HLA reference. After sorting, marking duplicates and indexing procedures, variant calling was performed using VarScan in tumor-only mode. An additional filter was applied to prioritize high quality calls (present in both read-mates) with a coverage>20 reads (for the reference base) and 4 reads (for the alternative allele). A further in-house developed java-based filter, exploiting the multi-alignment files provided by the HLA-IPD/IMGT database, was adopted to retain only non-polymorphic calls. Topographical and functional annotations were then performed on both coding and non-coding variants.

Allelic loss was imputed computing the number of reads covering each called heterozygous allele within a given locus according to the following formula:

$$Log2\frac{Ci}{(\Sigma Ci, Cz)/2}$$

Ci and Cz represent the read coverage for each homologous allele. For structurally-similar alleles, we included an adjustment taking into account sequence variation defined as “Variant coverage”, directly computed by NovoHLA pipeline.

All Log2 ratios <-1.5 were retained as confident allelic loss, based on a previous internal validation study on 234 healthy controls.

**Statistics.** All analyses (descriptive statistics) and data visualization were generated using the statistical computing environment R (4.0.0 R Core Team, R Foundation for Statistical Computing, Vienna, Austria), Excel Microsoft Office 365 and GraphPad Prism (8.4.0).

**Supplemental Table 1. Diagnostic targeted sequencing panel of The Cleveland Clinic Foundation.**

| *ABL1* | *CBL* | *DNMT3A* | *FLT3* | *IKZF1* | *KRAS* | *NPM1* | *PTEN* | *SF3B1* | *STAT3* | *WT1* |
| --- | --- | --- | --- | --- | --- | --- | --- | --- | --- | --- |
| *ASXL1* | *CDKN2A* | *EED* | *GATA1* | *JAK2* | *LUC7L2* | *NRAS* | *PTPN11* | *SH2B3* | *STAT5B* | *ZRSR2* |
| *BCOR* | *CEBPA* | *ETKN1* | *GATA2* | *JAK3* | *MPL* | *PHF6* | *RAD21* | *SMC1A* | *SUZ12* |  |
| *BCORL1* | *CSF3R* | *ETV6* | *GNAS* | *KDM6A* | *MYD88* | *PIGA* | *RIT1* | *SMC3* | *TET2* |  |
| *BRAF* | *CUX1* | *EZH2* | *IDH1* | *KIT* | *NF1* | *PPM1D* | *RUNX1* | *SRSF2* | *TP53* |  |
| *CALR* | *DDX41* | *FBXW7* | *IDH2* | *KMT2A* | *NOTCH1* | *PRPF8* | *SETBP1* | *STAG2* | *U2AF1* |  |

**Supplemental Figure 1. Bio-informatic pipeline for HLA mutation detection.**

**
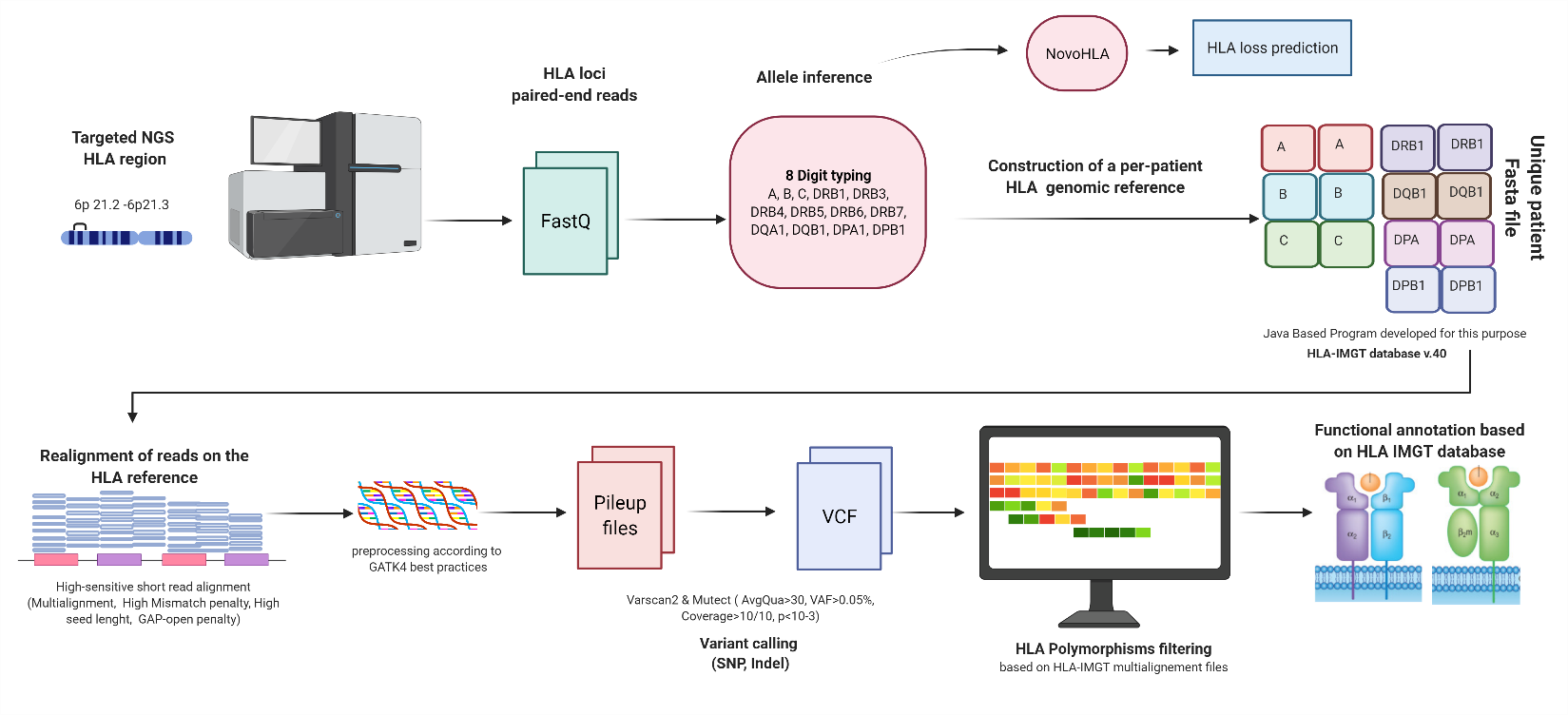
**

**References**

1. Gurnari C, Graham AC, Efanov A, Pagliuca S, Durrani J, Awada H, et al. Frequency and perturbations of various peripheral blood cell populations before and after eculizumab treatment in paroxysmal nocturnal hemoglobinuria. Blood Cells Mol Dis. 2020;87:102528.

2. Gurnari C, Pagliuca S, Patel BJ, Awada H, Kongkiatkamon S, Terkawi L, et al. Implication of PIGA genotype on erythrocytes phenotype in Paroxysmal Nocturnal Hemoglobinuria. Leukemia. 2021.

3. de Latour RP, Mary JY, Salanoubat C, Terriou L, Etienne G, Mohty M, et al. Paroxysmal nocturnal hemoglobinuria: natural history of disease subcategories. Blood. 2008;112(8):3099-106.

4. Sutherland DR, Illingworth A, Keeney M, Richards SJ. High-Sensitivity Detection of PNH Red Blood Cells, Red Cell Precursors, and White Blood Cells. Current Protocols in Cytometry. 2015;72(1):6.37.1-6..29.

5. Illingworth A, Marinov I, Sutherland DR, Wagner-Ballon O, DelVecchio L. ICCS/ESCCA Consensus Guidelines to detect GPI-deficient cells in Paroxysmal Nocturnal Hemoglobinuria (PNH) and related Disorders Part 3 – Data Analysis, Reporting and Case Studies. Cytometry Part B: Clinical Cytometry. 2018;94(1):49-66.

6. Li MM, Datto M, Duncavage EJ, Kulkarni S, Lindeman NI, Roy S, et al. Standards and Guidelines for the Interpretation and Reporting of Sequence Variants in Cancer: A Joint Consensus Recommendation of the Association for Molecular Pathology, American Society of Clinical Oncology, and College of American Pathologists. J Mol Diagn. 2017;19(1):4-23.

7. Nagata Y, Makishima H, Kerr CM, Przychodzen BP, Aly M, Goyal A, et al. Invariant patterns of clonal succession determine specific clinical features of myelodysplastic syndromes. Nature Communications. 2019;10(1):5386.

8. Nagata Y, Zhao R, Awada H, Kerr CM, Mirzaev I, Kongkiatkamon S, et al. Machine learning demonstrates that somatic mutations imprint invariant morphologic features in myelodysplastic syndromes. Blood. 2020;136(20):2249-62.

9. Awada H, Durmaz A, Gurnari C, Kishtagari A, Meggendorfer M, Kerr CM, et al. Machine Learning Integrates Genomic Signatures for Subclassification Beyond Primary and Secondary Acute Myeloid Leukemia. Blood. 2021.

10. Hirsch CM, Nazha A, Kneen K, Abazeed ME, Meggendorfer M, Przychodzen BP, et al. Consequences of mutant TET2 on clonality and subclonal hierarchy. Leukemia. 2018;32(8):1751-61.

11. Hirsch CM, Przychodzen BP, Radivoyevitch T, Patel B, Thota S, Clemente MJ, et al. Molecular features of early onset adult myelodysplastic syndrome. Haematologica. 2017;102(6):1028-34.

12. Clemente MJ, Przychodzen B, Hirsch CM, Nagata Y, Bat T, Wlodarski MW, et al. Clonal PIGA mosaicism and dynamics in paroxysmal nocturnal hemoglobinuria. Leukemia. 2018;32(11):2507-11.
